# Effects of Different Vegetation Types on Soil Hydrological Properties, Understory Diversity, and Water Conservation Capacity in the Horqin Sandy Land, China

**DOI:** 10.3390/plants15192967

**Published:** 2026-09-29

**Authors:** Jiahui Yao, Ruimin Liu, Hejun Zuo, Chenguang Wang, Xinghua Zhao, Jie Feng, Min Yan, Yang Xu

**Affiliations:** 1 Inner Mongolia Key Laboratory of Aeolian Physics and Desertification Control Engineering, College of Desert Control Science and Engineering, Inner Mongolia Agricultural University, Hohhot 010018, China; yjh00345@126.com (J.Y.); nmglrm@126.com (R.L.);; 2 Tongliao Institute of Forestry Sciences, Tongliao 028000, China

**Keywords:** northern sand prevention belt, Horqin Sandy Land, species diversity, water conservation capacity

## Abstract

The Northern Sand Prevention Belt is an important ecological security barrier in China. It is also one of the regions most severely affected by wind-eroded desertification. In this study, to investigate differences in soil properties among different vegetation types in sandy areas, a typical vegetation restoration area in the Horqin Sandy Land was selected. Widely distributed natural forests of *Ulmus pumila*, *Populus* plantations and *Pinus sylvestris* var. *mongolica* plantations were the study objects, with bare sandy land as the control. Understory herbaceous plant diversity and soil hydrological and physical properties were analysed, and Pearson correlation analysis, principal component analysis and the fuzzy membership function method were employed to comprehensively evaluate the soil water conservation function with respect to different vegetation types. The results showed that soil hydrological and physical properties differed significantly among the vegetation types. The *U. pumila* forest exhibited relatively low soil bulk density (1.53 g·cm^−3^) and high total porosity (32.42%) in the deep-soil layer (80–100 cm), indicating a strong capacity for deep-soil water storage. The *Populus* plantation showed the greatest soil improvement in the surface layer, with the highest capillary water-holding capacity and field water-holding capacity, reaching 17.82% and 16.05%, respectively. The *P. sylvestris* var. *mongolica* plantation had relatively high deep-soil bulk density (1.60 g·cm^−3^) and a pronounced decline in water-holding capacity. The unvegetated sandy land had the lowest capillary water-holding capacity (15.09%) and very poor water-retention capacity. Understory herbaceous plant diversity was highest in the *P. sylvestris* var. *mongolica* plantation and lowest in the *U. pumila* forest, and herbaceous plant diversity was significantly correlated with soil capillary porosity and capillary water-holding capacity. This comprehensive evaluation indicated that the poplar and *P. sylvestris* var. *mongolica* plantations had greater soil water conservation capacity than the natural *U. pumila* forest and unvegetated sandy land, with the tree–herbaceous vegetation structure exhibiting stronger synergistic characteristics and relatively favourable soil moisture conditions. These findings suggest that greater attention should be given to tree–herbaceous synergistic configurations in vegetation restoration in the Northern Sand Prevention Belt, while tree species selection should be optimised according to local conditions to enhance regional soil water conservation capacity.

## 1. Introduction

The Northern Sand Prevention and Desertification Control Belt is a major national ecological security barrier in northern China, playing an important role in windbreak and sand fixation, soil and water conservation, biodiversity conservation and the maintenance of regional ecological security [1]. It is of great significance for maintaining social stability and promoting economic development [2]. The Horqin Sandy Land is located in the semi-arid agro-pastoral ecotone of northern China and constitutes a major component of the Northern Sand Prevention and Desertification Control Belt [3]. Influenced by climate change and human activity [4], the region has experienced degradation of native vegetation and land desertification, resulting in a highly fragile ecological environment [5]. In recent years, China’s government has implemented a series of ecological restoration projects, including the Three-North Shelterbelt Program, the Natural Forest Protection Program, the Grain for Green Program and the Beijing–Tianjin Sandstorm Source Control Project. These have effectively protected and restored ecosystems [6], significantly increased regional vegetation cover and improved the ecological environment to some extent [7]. However, the Horqin Sandy Land receives relatively low precipitation with an uneven intra-annual distribution, and its soils are predominantly aeolian sandy soils with low water-holding capacity. Consequently, soil moisture remains a key limiting factor for vegetation restoration and the enhancement of ecosystem functioning [8]. Therefore, elucidating differences in soil water retention and water conservation functions among different vegetation restoration patterns is of great significance for enhancing ecosystem stability and consolidating achievements in sand prevention and desertification control in the Horqin Sandy Land.

Vegetation restoration is an important pathway for reversing land degradation and rebuilding healthy ecosystems. It can promote understory herbaceous growth [9] and improve soil physicochemical properties [10], and thereby influence the water conservation capacity of ecosystems. Previous studies have shown that vegetation restoration can reduce soil bulk density, improve soil pore structure and enhance soil water-holding capacity [11]. In the Horqin Sandy Land, relevant studies have also shown that vegetation restoration can significantly alter soil moisture, soil organic matter and nutrient status, thereby enhancing soil water retention capacity [12]. However, the extent of this improvement varies among vegetation restoration patterns. Different tree species can exert distinct effects on soil water dynamics due to differences in growth characteristics, root distribution and water-use strategies [13]. For example, some plantations can improve soil structure and enhance soil water retention by increasing surface cover, litter inputs and root-mediated effects [14], whereas the high transpiration and water consumption of fast-growing tree species may lead to soil moisture deficits in deeper soil layers [15]. Therefore, under the semi-arid conditions of the Horqin Sandy Land, whether vegetation restoration can improve soil water-holding capacity and whether different restoration patterns exert distinct hydrological effects remain to be further compared and validated.

In addition to woody vegetation, herbaceous plant communities are an important component linking vegetation restoration to soil water dynamics. The herbaceous layer plays a critical role in maintaining community structural stability, facilitating nutrient cycling and energy flow, and promoting soil and water conservation [16]. Herbaceous plant diversity not only reflects differences in plant community structure, composition and habitat conditions, but also serves as an important indicator for assessing ecosystem functioning and stability [17]. Previous studies have shown that higher plant diversity can improve resource-use efficiency and enhance ecosystem stability [18].

Herbaceous plants can influence soil structure and water retention processes through root-mediated soil stabilisation, ground cover and litter inputs, thereby playing an important role in soil water conservation [19]. Previous studies have indicated that herbaceous plant diversity is not only an important indicator of the ecological effects of vegetation restoration, but it also plays a key regulatory role in soil water retention. However, studies on vegetation restoration in the Horqin Sandy Land have focused primarily on vegetation cover, soil nutrients, soil organic carbon and vegetation growth; relatively little attention has been paid to the relationship between herbaceous plant diversity and soil water-holding properties. In particular, the synergistic relationship and underlying mechanisms between herbaceous plant diversity and soil water-holding properties remain poorly understood, and related research is limited.

Vegetation restoration in the Horqin Sandy Land has primarily relied on *Populus*, *Pinus sylvestris* var. *mongolica* and *Ulmus pumila* as the dominant tree species, resulting in the establishment of extensive shelterbelt systems [20]. These vegetation types differ markedly in tree species composition, stand structure and water-use strategies, which in turn affect herbaceous plant community composition and soil water retention capacity [21,22,23]. Water potential is a key parameter describing water distribution within the soil–plant–atmosphere continuum (SPAC). Differences among tree species in water potential regulation and water-use strategies provide an important basis for understanding interspecific differences in water requirements. Previous studies have shown that major afforestation tree species in the Horqin Sandy Land exhibit distinct water-use strategies: *P. sylvestris* var. *mongolica*— characterised by a well-developed cuticle, numerous small stomata and a relatively low specific leaf area—exhibits relatively high water-use efficiency and adopts a relatively conservative water-use strategy [24]. In contrast, *U. pumila* exhibits higher stomatal conductance and specific leaf area but relatively lower water-use efficiency [25]. Native and non-native tree species also differ in their sources of water [26].

Other interspecific differences in water-related traits may further contribute to divergent soil moisture dynamics among different vegetation restoration patterns. For example, differences in canopy structure can influence soil organic matter and pore structure by altering understory light availability and the redistribution of precipitation [27], while differences in root distribution and water-use patterns may alter the vertical distribution of soil moisture [28]. Comparing herbaceous plant diversity and soil hydrological properties among different vegetation restoration types can help clarify how different restoration patterns affect herbaceous communities and soil water dynamics. Specifically, comparing *U. pumila* forests, *Populus* plantations and *P. sylvestris* var. *mongolica* plantations with bare sandy land can reveal ecohydrological differences among restoration with native tree species, fast-growing plantations, coniferous plantations and degraded conditions lacking vegetation cover, thereby providing more targeted evidence for identifying suitable vegetation restoration patterns.

Although previous studies have revealed the effects of vegetation restoration on soil properties or plant diversity, the patterns of coordinated variation between herbaceous plant diversity and soil water-holding properties with respect to different vegetation types, as well as comprehensive, multi-indicator assessments of soil water conservation capacity, remain poorly understood. A single soil indicator is insufficient to comprehensively characterise the water conservation capacity of an ecosystem, whereas integrating soil physical properties, water-holding characteristics and herbaceous plant diversity can provide a more comprehensive assessment of the ecohydrological effects of different vegetation restoration patterns.

Accordingly, the present study aimed to: clarify the effects of different vegetation restoration types on herbaceous plant diversity and soil water-holding properties; reveal the synergistic relationship between herbaceous plant diversity and soil water-holding properties; and evaluate the overall soil water conservation capacity of different vegetation restoration types and compare their ecohydrological effects and differences. The study was conducted in a typical vegetation restoration area of the Horqin Sandy Land within the Northern Sand Prevention and Desertification Control Belt. *U. pumila* natural forest, poplar plantation, and *P. sylvestris* var. *mongolica* plantation were selected as the study objects, with bare sandy land serving as the control. Vegetation community characteristics and soil physicochemical properties were investigated and measured in relation to different vegetation types. The relationship between herbaceous plant diversity and soil water-holding properties was analysed, a comprehensive assessment system for soil water conservation capacity was established, and the ecohydrological differences among the vegetation types were compared.

Based on previous studies and the ecohydrological characteristics of the Horqin Sandy Land, the following hypotheses were proposed:

(1) Herbaceous plant diversity is positively correlated with soil water-holding properties, such that higher herbaceous plant diversity promotes soil water retention by increasing root and litter inputs, improving soil structure and enhancing ground cover.

(2) Different vegetation restoration types exhibit different overall water conservation capacities, with restoration types characterised by more suitable stand structures and stronger soil–vegetation synergistic interactions expected to show higher comprehensive evaluation values.

## 2. Result

### 2.1. Variation Characteristics of Soil Bulk Density and Porosity

As shown in Figure 1, the mean soil bulk density across the study area was approximately 1.57 g·cm^−3^, with some differences among vegetation types. In the JY plot, the mean soil bulk density was approximately 1.58 g·cm^−3^ and decreased with increasing soil depth. Soil bulk density in the 0–40 cm soil layer was significantly higher than in the deeper soil layers (*p* < 0.05). In contrast, soil bulk density in the ZZ, YS and CK plots gradually increased with increasing soil depth. In the ZZ plot, soil bulk density in the surface layer was only 1.51 g·cm^−3^, significantly lower than in the other soil layers (*p* < 0.05). In the YS plot, no significant differences in soil bulk density were observed among the soil layers, with values remaining around 1.57 g·cm^−3^. Significant differences were observed among soil layers in the CK plot (*p* < 0.05), with soil bulk density peaking at 1.59 g·cm^−3^ at 80–100 cm, which was significantly higher than in the other soil layers (*p* < 0.05). Among the different plots, soil bulk density in the surface layer of JY was significantly higher than in the other plots (*p* < 0.05). However, in the deeper soil layers, soil bulk density in the ZZ plot was significantly higher than in the other vegetation restoration plots (*p* < 0.05), whereas JY had the lowest value at only 1.53 g·cm^−3^.

The variation in total soil porosity was generally opposite to that of soil bulk density. In the JY plot, total porosity gradually increased with increasing soil depth, with a mean value of 32.02%. Total porosity in the deeper soil layers was significantly higher than in the surface layer (*p* < 0.05). In contrast, ZZ, YS and CK showed a decreasing trend in total porosity with increasing soil depth. Total porosity was significantly higher in the surface layer than in the bottom soil layer (*p* < 0.05). Among the different plots, total porosity in the surface layers of CK and ZZ was significantly higher than that of YS and JY (*p* < 0.05). In contrast, in the deeper soil layers, total porosity was significantly higher in JY and YS than that in CK (*p* < 0.05).

Capillary porosity generally showed an increasing trend with increasing soil depth. In the JY plot, capillary porosity varied relatively little among soil depths. There were no significant differences among the 0–60 cm soil layers (*p* > 0.05), but these values were significantly higher than those in the bottom soil layer (*p* < 0.05). In the ZZ plot, capillary porosity was relatively high in the surface layer and significantly higher than in the other soil layers (*p* < 0.05). Among the different plots, CK had the lowest soil capillary porosity at only 23.34%, and showed a pronounced decline below 60 cm, with values significantly lower than those of the other vegetation types (*p* < 0.05).

The variation in non-capillary porosity was generally opposite to that of capillary porosity, showing a gradual increase with increasing soil depth. The mean non-capillary porosity in CK was approximately 9.37%, higher than in the other plots, whereas YS had the lowest value at 4.63%. Among the different plots, non-capillary porosity in CK was significantly higher than in the other vegetation types across all soil layers (*p* < 0.05).

### 2.2. Soil Water-Holding Capacity

Soil water-holding capacity is one of the major indicators for evaluating the soil water conservation function. It is jointly determined by soil pore characteristics and soil depth. As shown in Figure 2, the maximum water-holding capacity, capillary water-holding capacity, field capacity and soil water content differed to some extent among the different plots.

With increasing soil depth, the maximum water-holding capacity in the JY plot showed an increasing trend, although the differences among soil layers were not significant (*p* > 0.05). In contrast, the maximum water-holding capacity in the ZZ, YS and CK plots decreased with increasing soil depth. The largest decline was observed in ZZ, where it decreased from 23.83% in the surface layer to 21.08%. The maximum water-holding capacity in CK remained relatively stable across soil layers, although significant differences were observed among soil layers (*p* < 0.05). Capillary water-holding capacity and field capacity showed similar patterns, both decreasing with increasing soil depth. Among the different plots, YS had the highest overall capillary water-holding capacity and field capacity, reaching 18.01% and 16.05%, respectively, whereas bare sandy land had the lowest values, at 12.54% and 15.09%, respectively. The capillary water-holding capacity of CK and YS differed significantly in the deeper soil layers (*p* < 0.05), whereas significant differences in field capacity were observed across all soil layers (*p* < 0.05). In the ZZ plot, both capillary water-holding capacity and field capacity decreased markedly below 20 cm, and their mean values within the 20–100 cm soil layer were lower than those in JY and YS.

Soil water content differed markedly among vegetation types. JY had the highest mean soil water content (5.41%). This was significantly higher than that in CK (*p* < 0.05) but did not differ significantly from the mean soil water content in ZZ and YS (*p* > 0.05). With increasing soil depth, soil water content in the JY forest gradually decreased, reaching 3.68% at 80–100 cm, which was significantly lower than that in ZZ and YS (*p* < 0.05). CK showed a similar pattern, with soil water content decreasing from 5.20% to 3.72%, and a significant difference was observed between the surface and bottom soil layers (*p* < 0.05). In YS, soil water content gradually decreased with increasing depth within the 0–60 cm soil layer, followed by an increase and then a subsequent decrease below 60 cm; however, the differences among soil layers were not significant (*p* > 0.05). In contrast, ZZ showed an overall increasing trend in soil water content with increasing soil depth. Soil water content was only 3.68% in the surface layer, increased to 5.67% at greater depths, and then declined slightly below 80 cm to 5.01%.

### 2.3. Characteristics of Understory Herbaceous Diversity

The vegetation community diversity indices differed markedly among the different plots (Table 1). Among the vegetation restoration plots, the ZZ plot had the highest Shannon–Wiener diversity index (2.59 ± 0.01). This was significantly higher than that of JY (*p* < 0.05) but did not differ significantly from that of YS (*p* > 0.05). The Simpson dominance index showed a similar pattern, with the highest value observed in ZZ (0.52 ± 0.02). This was significantly higher than that of JY (0.49 ± 0.20; *p* < 0.05) but did not differ significantly from that of YS (0.51 ± 0.02; *p* > 0.05). The Margalef richness index followed a pattern similar to that of the Shannon–Wiener index, with the highest value in ZZ (4.15 ± 0.13), followed by YS (3.89 ± 0.09) and JY (3.34 ± 0.64). The Margalef richness index in ZZ was significantly higher than that in JY (*p* < 0.05) but did not differ significantly from that in YS (*p* > 0.05). Overall, the herbaceous communities beneath the *ZZ* and YS plantations exhibited relatively higher species richness and diversity, whereas herbaceous diversity beneath the natural JY open woodland was relatively low.

### 2.4. Correlations Between Soil Properties and Herbaceous Diversity

Pearson correlation analysis was performed on 12 indicators of soil physicochemical properties and herbaceous plant diversity with respect to different vegetation restoration types, with the results shown in Figure 3. The indicators exhibited varying degrees of correlation, with strong associations observed between soil pore structure and water-holding capacity. Soil bulk density was significantly negatively correlated with capillary porosity and capillary water-holding capacity (*p* ≤ 0.05), whereas total porosity was significantly positively correlated with capillary water-holding capacity (*p* ≤ 0.05). Capillary porosity was significantly positively correlated with maximum water-holding capacity and capillary water-holding capacity (*p* ≤ 0.05) but was highly significantly negatively correlated with non-capillary porosity (*p* ≤ 0.01). Maximum water-holding capacity was highly significantly positively correlated with capillary water-holding capacity (*p* ≤ 0.01). Non-capillary porosity, capillary water-holding capacity and field capacity showed no significant correlations with the other indicators. Soil water content was significantly negatively correlated with the Shannon–Wiener diversity index (*p* ≤ 0.05) and highly significantly negatively correlated with the Simpson dominance index (*p* ≤ 0.01). The Shannon–Wiener diversity index was highly significantly positively correlated with both the Simpson dominance index and the Margalef richness index (*p* ≤ 0.01), while the Simpson dominance index was significantly positively correlated with the Margalef richness index (*p* ≤ 0.05).

### 2.5. Principal Component Analysis of Soil Properties and Herbaceous Diversity

As shown in Table 2 and Table 3, the eigenvalues, variance contribution rates and cumulative variance contribution rates of the principal components were calculated. The eigenvalues of the first three principal components were all greater than 1. The contribution rates of PC1, PC2 and PC3 were 45.39%, 28.48% and 10.92%, respectively, with a cumulative contribution rate of 84.78%. This indicated that these three principal components adequately explained the overall variation in soil water conservation function indicators among the different vegetation restoration types. PC1, with the highest contribution rate (45.39%), was primarily dominated by soil bulk density, total porosity, capillary porosity, capillary water-holding capacity, field capacity and soil water content, all of which had relatively high loadings. Thus, PC1 represented the overall status of soil pore structure and water storage and retention capacity. PC2 accounted for 28.45% of the total variance, with the Shannon–Wiener diversity index, Simpson dominance index and Margalef richness index showing relatively high loadings. Thus, PC2 primarily reflected the regulatory effects of plant community species composition and diversity on soil water conservation function. PC3 accounted for 10.92% of the total variance and was mainly influenced by non-capillary porosity, representing the capacity for temporary water storage and rapid drainage and reflecting the soil’s infiltration and drainage regulation function. The three principal components were used for the subsequent comprehensive evaluation of soil water conservation function.

### 2.6. Comprehensive Evaluation of Water Conservation Capacity in Relation to Different Vegetation

#### Restoration Types

Based on the results of the principal component analysis, the membership function values of PC1–PC3 were further calculated for the three vegetation restoration types, and the comprehensive evaluation index (D value) was obtained. The results are presented in Table 4. The D values of the three vegetation restoration types ranged from 0.48 to 0.82, with higher D values indicating stronger ecohydrological synergy. The three common vegetation restoration types in the Horqin Sandy Land were ranked and classified according to their D values. There were marked differences in soil water conservation function among the different vegetation restoration types. YS had the highest D value of 0.82, indicating the strongest soil water conservation capacity, followed by *ZZ*, with a D value of 0.74. The JY plot had a D value of 0.48, indicating a relatively lower overall capacity. CK, representing the initial stage of vegetation restoration, had the weakest soil water conservation capacity. Overall, the soil water conservation capacity of the different vegetation restoration types followed the order YS > ZZ > JY > CK.

## 3. Discussion

### 3.1. Differences in the Ameliorative Effects of Different Vegetation Types on Soil Hydrophysical Properties

Soil hydrological effects are important determinants of forest water conservation capacity, soil and water conservation, and vegetation growth and development [29]. Soil bulk density, porosity, water-holding capacity, permeability and water-stable aggregates can reflect differences in soil physical properties among different vegetation types [30]. Differences in the composition and decomposition status of litter layers, belowground root systems and soil development among vegetation types may result in significant differences in the effects of different vegetation restoration types on soil bulk density, porosity and water-holding capacity in the Horqin Sandy Land, with distinct vertical distribution patterns [31]. The evaluation results indicated that the YS and ZZ restoration types exhibited relatively strong ecological–hydrological synergy and could substantially improve the physical structure and water-holding capacity of aeolian sandy soils. However, the underlying mechanisms differed to some extent.

In the YS plot, soil bulk density increased with depth, while total porosity exhibited the opposite trend. Despite this vertical pattern, the YS plot recorded the highest capillary and field water-holding capacities among all treatments, suggesting superior soil water-retention ability. This may have been due to the characteristics of *Populus*, a fast-growing broadleaved species with a vigorous and rapidly renewing root system. Root growth, together with subsequent root mortality and decomposition, is known to create abundant biopores, which in turn enhance soil permeability and the pore structure critical for water retention. Indeed, poplar is a major species used in shelterbelt construction in northeastern China [32], and its positive effects on soil physical properties have been corroborated by vegetation restoration studies on the Songnen Plain [33]. However, with increasing soil depth, root activity gradually decreases, organic matter inputs are reduced, and the soil is subjected to prolonged gravitational compaction, resulting in increased bulk density in the deeper soil layers [34]. In the present study, the YS plot maintained relatively uniform water-holding capacity across soil layers, which may have been associated with its relatively even root distribution and stable capillary pore structure. Consequently, the YS restoration type exhibited the strongest soil water conservation function among the vegetation restoration types in the study area.

Although the ZZ plot exhibited relatively high total porosity and relatively high herbaceous plant diversity, its maximum water-holding capacity and capillary water-holding capacity showed a pronounced decreasing trend with increasing soil depth. This indicated that its soil water-holding capacity may be concentrated primarily in the surface soil, whereas the water-retention capacity of deeper soil layers was relatively weak. Previous studies have indicated that the root system of *P. sylvestris* var. *mongolica* may be relatively concentrated in shallow soil layers, while the high lignin content of its needle litter may contribute to relatively slow decomposition [35], thereby limiting the input of organic matter into deeper soil layers and its contribution to soil structural improvement. However, these proposed mechanisms require further validation through direct measurements of root distribution and litter decomposition. The present study showed that soil water-holding capacity in the ZZ plot decreased in the deeper soil layers, suggesting that, under the experimental conditions of this study, these plantations may have relatively limited water-retention capacity in deep-soil layers.

Although the JY plot had a slightly lower comprehensive evaluation score than the *YS* and *ZZ* plots, it exhibited the highest soil water content. Additionally, its maximum water-holding capacity and total porosity increased with increasing soil depth, indicating a strong capacity for deep-soil water storage. In contrast to the plantations, *U. pumila*, a typical native tree species of the Horqin Sandy Land, has a well-developed deep root system and strong drought adaptation capacity [36]. Over the long term, the root system of *U. pumila* continuously extends into deeper soil layers and forms numerous root channels. Following root mortality and decomposition, these channels are further transformed into stable biopores, thereby improving deep-soil structure and increasing soil porosity and water storage space in deeper soil layers [37,38]. Furthermore, the study area is predominantly characterised by naturally occurring sparse forest communities. The surface soil has been subjected to long-term aeolian sediment deposition, livestock trampling and the dense distribution of fine roots in the upper soil layer, resulting in relatively compact surface soil and consequently higher soil bulk density [39]. In contrast, deeper soil layers are more strongly influenced by root penetration and biotic disturbance, resulting in a vertical distribution pattern characterised by decreasing soil bulk density and increasing soil porosity with increasing soil depth [40]. Meanwhile, the relatively high porosity of the deeper soil layers facilitates the downward infiltration and storage of precipitation, resulting in significantly higher deep-soil water content than that observed in cropland and grassland [41]. Therefore, although the *JY* had slightly lower capillary water-holding capacity and field water-holding capacity than the YS, it exhibited a stronger capacity for deep-soil water storage, conferring a distinct advantage in maintaining long-term ecosystem stability and drought resilience.

### 3.2. Characteristics of Understory Herbaceous Diversity and Its Synergistic Relationship with Soil Hydrological Functions

The understory herbaceous layer is an important component of terrestrial ecosystems. It is highly sensitive to environmental changes, making it a common indicator for assessing ecosystem stability and functional recovery [42]. The present study found that, among the four vegetation types, the ZZ plot exhibited the highest herbaceous plant diversity, significantly higher than the JY plot but not significantly different from that in the YS plot. These results indicate that plantations can significantly increase understory herbaceous species richness compared with natural forests, consistent with previous findings from plantation forests in the hilly region of the Loess Plateau [43]. This was possibly because the JY plot is subject to relatively intense grazing activity. Trampling and browsing by livestock may inhibit the establishment and growth of understory herbaceous plants [44], resulting in species loss and reduced herbaceous plant diversity [45]. In contrast, the ZZ plot has relatively wide tree spacing and a high proportion of canopy gaps, resulting in greater light availability in the understory that promotes the growth of understory herbaceous plants [46].

### 3.3. Optimisation of Water Conservation Function Among Different Vegetation Restoration Models Based on Comprehensive Evaluation

Soil hydrological effects influence forest water conservation capacity, soil and water conservation, and vegetation growth and development [47]. In the present study, soil bulk density was highest in the ZZ plot, followed by the JY and YS plots, and was lowest in the CK plot. In terms of soil water-holding capacity, the YS plot exhibited the highest capillary water-holding capacity, followed by the JY and ZZ plots, with the CK plot having the lowest value. For herbaceous plant diversity, the ZZ plot showed the highest diversity, followed by the YS and JY plots, with the CK plot showing the lowest diversity.

The comprehensive evaluation indicated that the *YS* plot had the highest water conservation capacity. This suggested that the *YS* plot had relatively appropriate tree spacing and stand density [48], which not only maintained the windbreak and sand-fixation function of the tree layer but also provided suitable light conditions for understory herbaceous vegetation. Additionally, the relatively high root biomass of poplars may contribute to more comprehensive improvements in soil physical properties [49] The relatively high understory herbaceous diversity contributed to the formation of a two-layer vegetation structure consisting of trees and herbaceous plants. The herbaceous root network may enhance soil water conservation by modifying the distribution of capillary pores, while tree roots promote soil loosening through root penetration. These complementary effects may act synergistically to improve the soil water conservation capacity. In contrast, the *ZZ* plot had a relatively low comprehensive evaluation score. Although it exhibited the highest herbaceous plant diversity, the deterioration of deep-soil structure and the decline in deep-soil water-holding capacity resulted in relatively weak overall ecological–hydrological functioning.

Previous studies have shown that, in semi-arid regions, *P. sylvestris* var. *mongolica* plantations may face a risk of soil moisture deficit due to an imbalance between water supply and demand [50]. However, this process exhibits strong regional variability and is influenced by factors such as stand age, stand density, precipitation conditions and forest management practices [51]. Therefore, under the plot conditions and evaluation indicator system adopted in the present study, the ZZ plot exhibited a relatively low overall soil water conservation capacity. This finding suggests that, when establishing *P. sylvestris* var. *mongolica* plantations under similar water availability conditions, particular attention should be given to their capacity to maintain deep-soil moisture, and their suitability should be evaluated comprehensively in conjunction with stand density, water availability and long-term monitoring results.

In summary, vegetation restoration practices in the Horqin Sandy Land and other similar semi-arid regions should place greater emphasis on the synergistic relationships among tree species, stand structure and the understory herbaceous layer. First, excessive reliance on monoculture plantations of a single tree species should be avoided, and tree species should be appropriately selected and configured according to regional precipitation, soil moisture conditions, stand density and forest management practices. For *P. sylvestris* var. *mongolica* plantations, particular attention should be paid to the maintenance of deep-soil moisture, and their ecological–hydrological effects should be evaluated in conjunction with long-term monitoring data rather than generalised or restricted solely on the basis of comprehensive evaluation results from a single period. Second, greater attention should be given to tree–herbaceous synergistic configurations. Maintaining relatively high herbaceous plant diversity and an appropriate stand structure may promote coordinated improvements in soil structure and water-retention capacity. Finally, the protection and appropriate management of natural sparse *U. pumila* forests should be strengthened to fully harness the potential advantages of this native tree species in maintaining regional soil moisture and ecosystem stability.

## 4. Conclusions

This study investigated soil hydrological and physical properties and understory herbaceous diversity with respect to three typical vegetation types in the Horqin Sandy Land. Their soil water conservation functions were analysed using principal component analysis and the fuzzy membership function method. The results showed that different vegetation restoration patterns significantly affected soil structure, water-holding capacity and herbaceous communities. The natural *U. pumila* forest exhibited strong deep-soil water storage capacity, while the *Populus* plantation showed superior soil capillary water-holding and field water-holding capacities. Although the *P. sylvestris* var. *mongolica* plantation supported relatively high herbaceous diversity, its deep-soil water-holding capacity was relatively weak. The unvegetated sandy land had the lowest water conservation function. Correlation analysis indicated a close relationship between soil capillary porosity and soil water-holding capacity. The comprehensive evaluation showed that the water conservation capacity of the four vegetation types followed the order *Populus* plantation > *P. sylvestris* var. *mongolica* plantation > *U. pumila* forest > bare sandy land. These findings suggest that, under the semi-arid sandy conditions of the study area and in similar regions, greater attention should be given to the complementary configuration of trees and herbaceous vegetation. Restoration tree species should be selected appropriately by considering regional water availability, stand structure and species adaptability to enhance the water conservation function of sandy-land ecosystems.

## 5. Materials and Methods

### 5.1. Study Area

The study area is in the interspersed sand dune–depression landscape in the southeastern part of the Horqin Sandy Land, with geographic coordinates ranging from 121°58′00″—123°20′00″ E and 42°39′52″—43°40′28″ N. Administratively, it is in Horqin Left Rear Banner, Tongliao City, Inner Mongolia Autonomous Region, China. The overview of the study area is shown in Figure 4. The region has a temperate continental monsoon climate characterised by distinct seasons and a synchronous occurrence of rainfall and heat. The mean annual precipitation is 375.3 mm, with most precipitation occurring from June to September, while the mean annual evaporation is 1800–2100 mm. The soils are predominantly aeolian sandy soils, which have relatively low water- and nutrient-holding capacities. The vegetation in the study area is primarily characterised by a *U. pumila* open woodland ecosystem, with *U. pumila* and *Ulmus macrocarpa* serving as the constructive and dominant species. Associated tree species include *Crataegus pinnatifida* and *Acer truncatum.* The shrub layer is mainly composed of *Caragana microphylla*, *Caragana korshinskii* and *Lespedeza davurica*, while the herbaceous layer primarily includes species such as *Setaria viridis*, *Artemisia scoparia*, *Cleistogenes serotina* and *Carex korshinskyi.* Overall, the vegetation in the study area exhibits typical characteristics of semi-arid open woodland–grassland ecosystems. Thus, it represents a typical region, suitable for studying vegetation restoration and associated ecosystem functions in the Horqin Sandy Land.

### 5.2. Experimental Sites Design

Three vegetation types, *U. pumila* forest (JY), poplar forest (YS), and *P. sylvestris* var. *mongolica* forest (ZZ), were selected as the study objects in the study area, with bare sandy land (CK) serving as the control. The *U. pumila* forest is a naturally occurring open woodland that has long been subject to relatively intensive livestock grazing and trampling. In contrast, the *Populus* and *P. sylvestris* var. *mongolica* forests are managed plantations with relatively limited livestock disturbance and stand ages of approximately 10−12 years. Stand age was estimated based on diameter at breast height, tree height and surveys of local forest management history. Differences in grazing intensity, afforestation density, stand density and canopy openness were recorded as background information in Table 5. Three independent large plots (50 m × 50 m) were established for each vegetation type as biological replicates (*n* = 3 per vegetation type), resulting in a total of 9 plots. Within each plot, three 1 m × 1 m herbaceous quadrats were arranged in a triangular pattern. The species composition, number of individuals, plant height, and other community characteristics of herbaceous plants within each quadrat were surveyed and recorded. A soil profile (1 m × 1 m × 1 m; length × width × depth) was excavated adjacent to each herbaceous quadrat, and undisturbed soil samples were collected from the 0−20, 20−40, 40−60, 60−80 and 80−100 cm soil layers to determine soil physical properties, including soil bulk density and porosity.

### 5.3. Determination of Soil Hydrological Effect Indicators

Following the Chinese Forestry Determination of Soil Moisture and Physical Properties of Forest Soils industry standard (LY/T 1215–1999), the ring-cylinder method was used to determine soil bulk density, total porosity, capillary porosity and non-capillary porosity, as well as soil gravimetric water content, maximum water-holding capacity, capillary water-holding capacity and field capacity [52].

(1) $$\mathrm{Soil}\;\mathrm{natural}\;\mathrm{water}\;\mathrm{content}\;(\%)=\frac{m_{0}-m_{4}}{m_{4}-m}\times 100$$

(2) $$\mathrm{Maximum}\;\text{water-holding}\;\mathrm{capacity}\;(\%)=\frac{m_{2}-m_{4}}{m_{4}-m}\times 100$$

(3) $$\mathrm{Capillary}\;\text{water-holding}\;\mathrm{capacity}\;(\%)=\frac{m_{2}-m_{4}}{m_{4}-m}\times 100$$

(4) $$\mathrm{Minimum}\;\text{water-holding}\;\mathrm{capacity}\;(\%)=\frac{m_{3}-m_{4}}{m_{4}-m}\times 100$$

(5) $$\mathrm{Soil}\;\mathrm{bulk}\;\mathrm{density}=\frac{m_{4}-m}{100}$$

(6) $$\mathrm{Total}\;\mathrm{porosity}=\frac{m_{1}-m_{4}}{100}$$

(7) $$\mathrm{Capillary}\;\mathrm{porosity}=\frac{m_{2}-m_{4}}{100}$$

Non-capillary porosity = total porosity − capillary porosity(8)

Here, *m*_0_ is the fresh weight of the ring sampler; *m*_1_ is the weight of the ring sampler after 24 h of immersion; *m*_2_ is the weight of the ring sampler after draining on a flat-bottomed tray covered with dry sand for 2 h; *m*_3_ is the weight of the ring sampler after further draining for 24 h; *m*_4_ is the weight of the ring sampler after oven-drying; and m is the dry weight of the ring sampler with filter paper.

### 5.4. Determination of Herbaceous Diversity

(1) Shannon–Wiener diversity index (*H*):
  $$H=-\sum_{i=1}^{s}P_{i}\ln P_{i}$$
(2) Simpson dominance index (*H*′):
  $$H^{\prime }=1-\sum_{i=1}^{s}P_{i}^{2}$$
(3) Pielou evenness index (*J*):
  J=H/lnS
(4) Margalef richness index (*D*):
  $$D=\frac{S-1}{\ln N}$$

Here, *S* is the total number of species in the quadrat and *P_i_* is the relative importance value of species *i*.

### 5.5. Data Processing and Analysis

Based on the measured soil hydrological effect indicators and vegetation community characteristics, the fuzzy membership function method was used to comprehensively evaluate the eco-hydrological synergistic enhancement capacity and water conservation capacity under different vegetation restoration approaches. The specific steps were as follows:

(1) Membership function value:
  $$u(X_{j})=\frac{\left(X_{j}-X_{\min}\right)}{\left(X_{max}-X_{min}\right)}$$
(2) Weight of each comprehensive indicator:
  $$W_{j}=\frac{V_{j}}{\sum_{j=1}^{m}V_{j}}$$
(3) Comprehensive evaluation value:
  $$D=\sum_{i=1}^{n}\left[u(X_{j})\times W_{j}\right]$$

Here, *u(X_j_)* is the membership function value of the *j*th indicator; *X_j_* is the relative value of the *j*th indicator; *X_max_* and *X_min_* are the maximum and minimum values, respectively, of the corresponding indicator among all indicators included in the evaluation; *W_j_* is the weight of the *j*th indicator among all indicators; *V_j_* represents the contribution rate of the *j*th indicator; and *D* is the comprehensive evaluation value of the synergistic improvement of ecohydrological functions and water conservation capacity with respect to different vegetation restoration types.

## Figures and Tables

**Figure 1:**
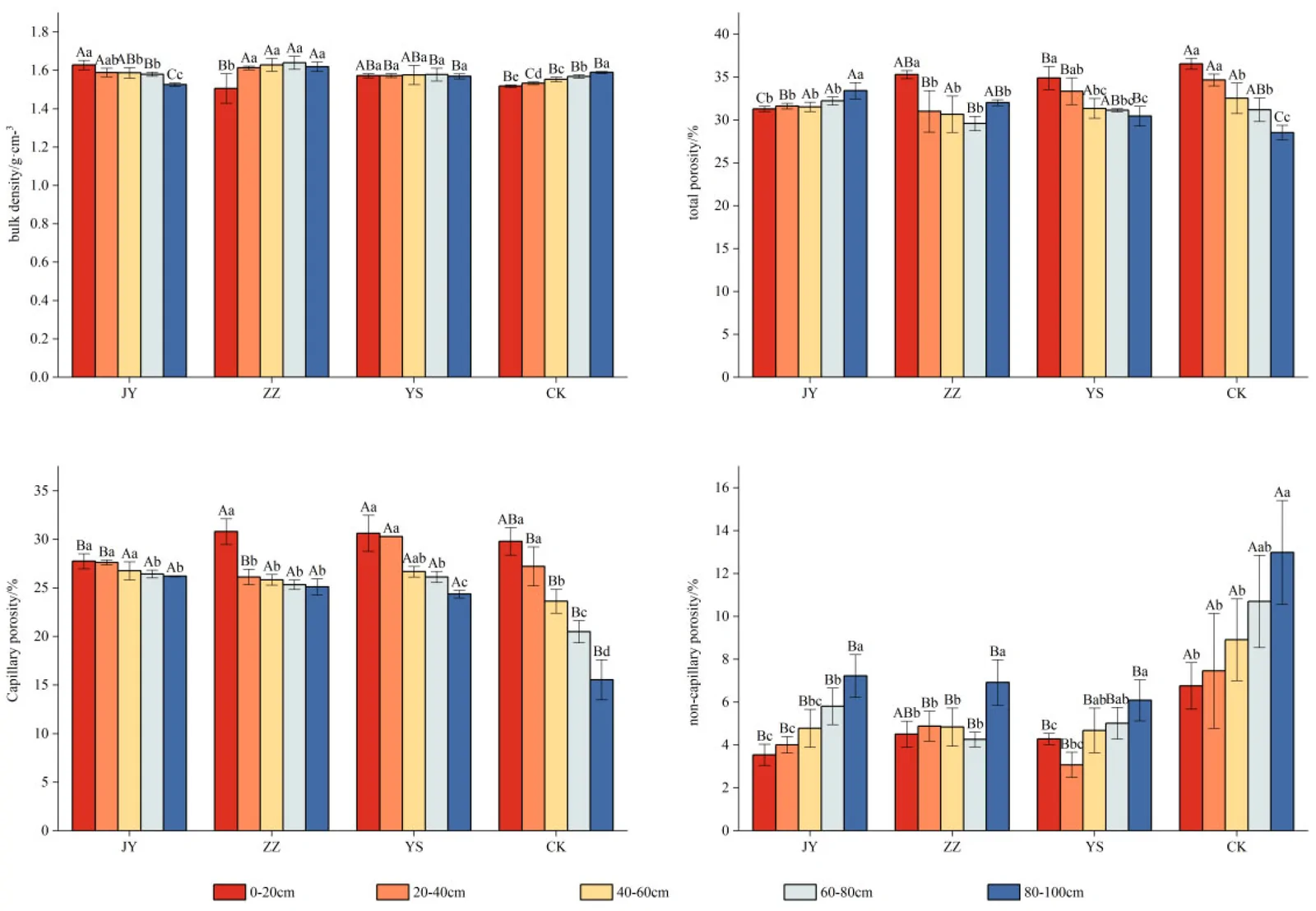
Variation characteristics of soil bulk density and porosity in relation to different vegetation restoration types. Note: Different lowercase letters indicate significant differences among soil layers within the same plot (*p* < 0.05), whereas different uppercase letters indicate significant differences among plots within the same soil layer (*p* < 0.05).

**Figure 2:**
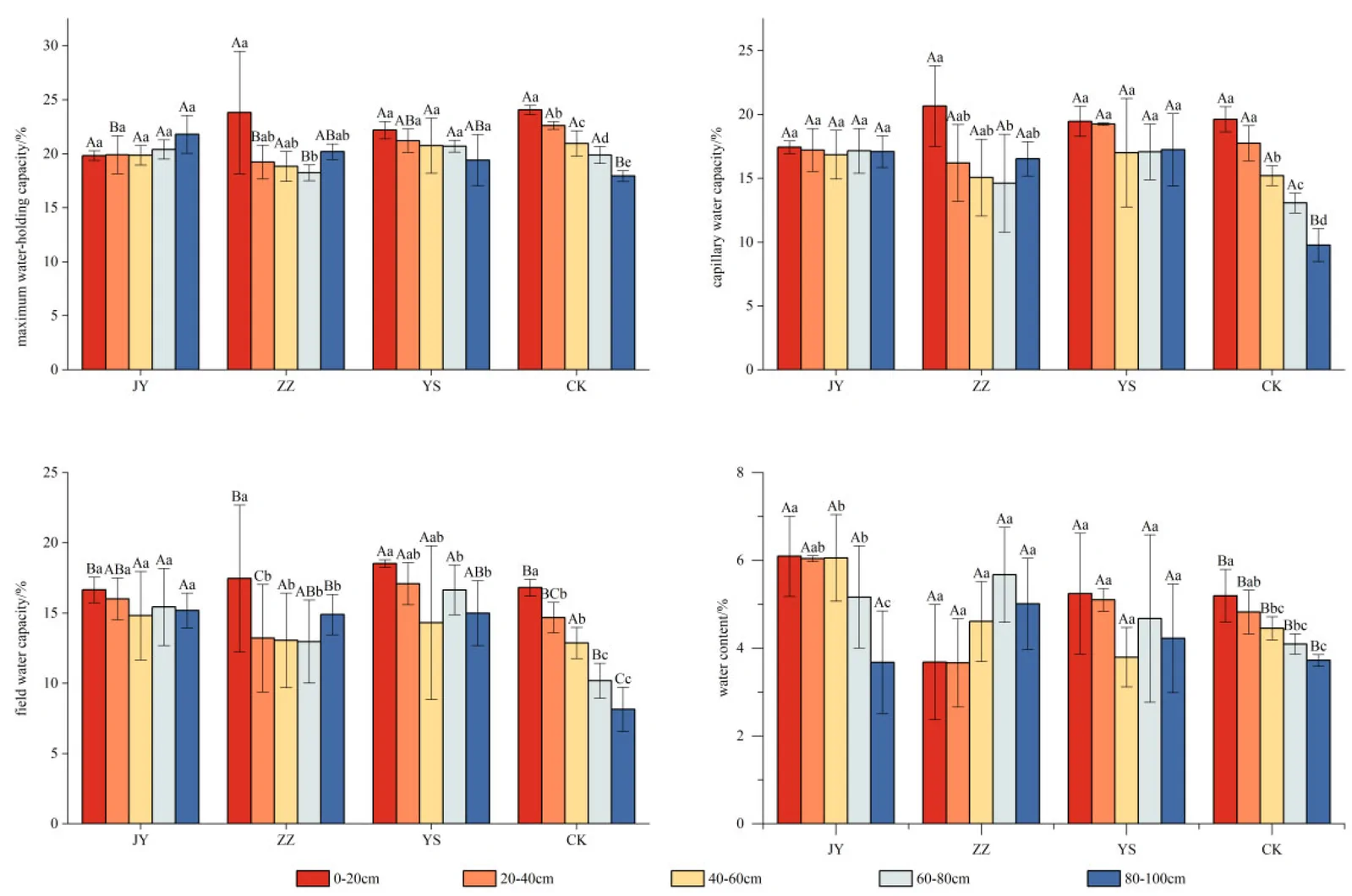
Soil water-holding capacity with respect to different vegetation restoration types. Note: Different lowercase letters indicate significant differences among soil layers within the same plot (*p* < 0.05), whereas different uppercase letters indicate significant differences among plots within the same soil layer (*p* < 0.05).

**Figure 3:**
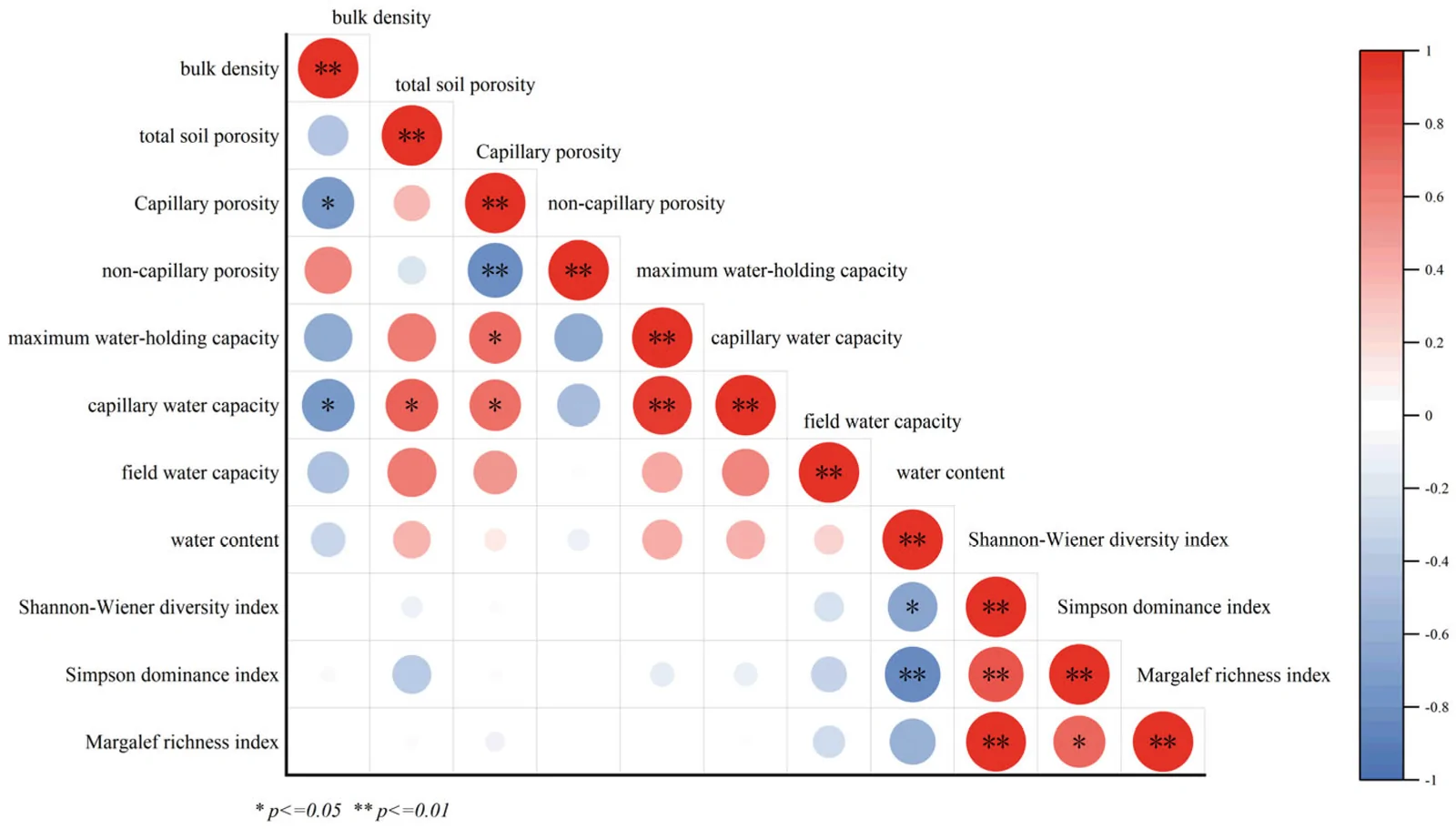
Correlation analysis of various indicators in relation to different vegetation types.

**Figure 4:**
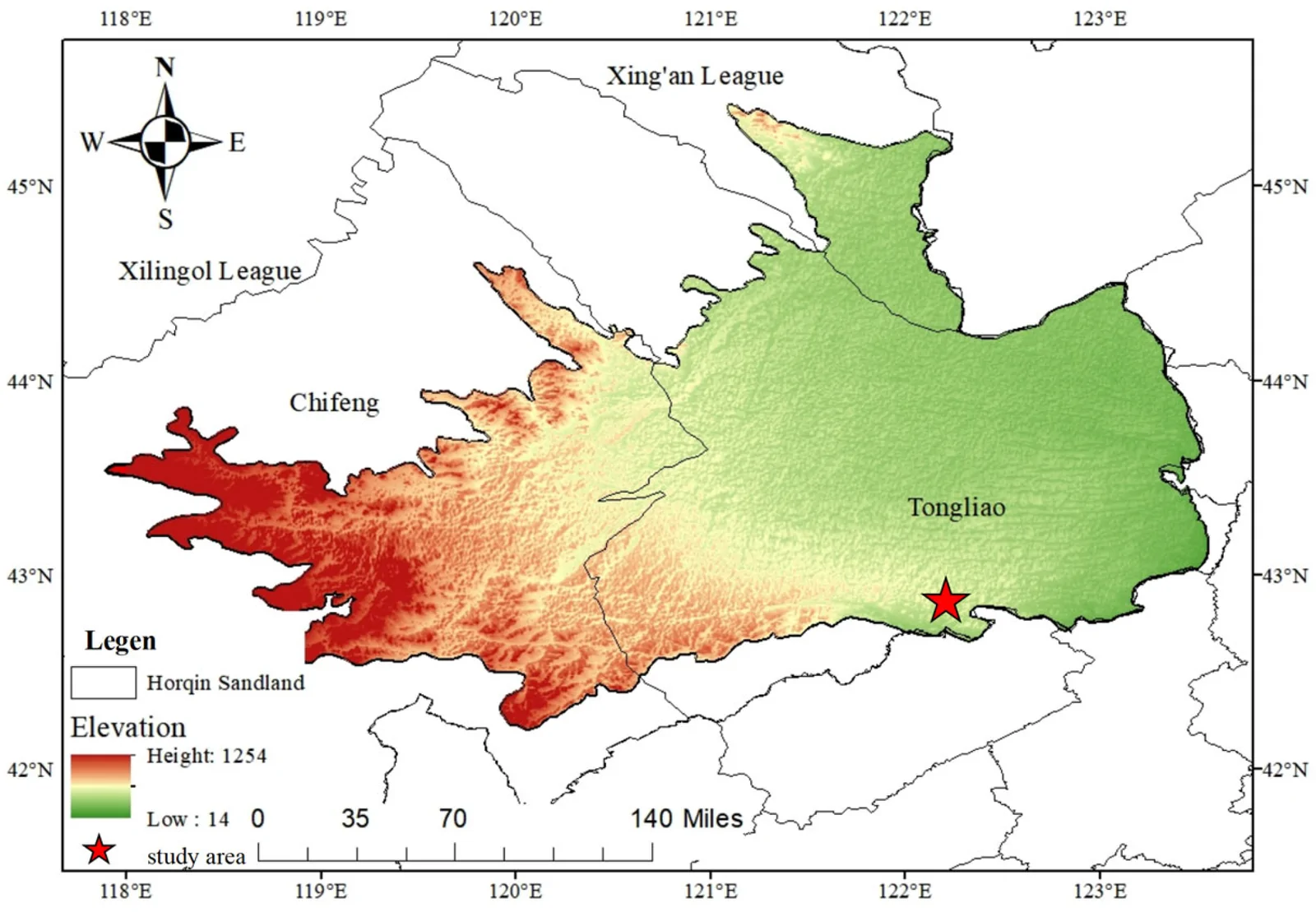
Overview of the study area.

**Table 1:** Understory herbaceous diversity in relation to different vegetation restoration types.

Plot	Shannon–Wiener Diversity Index	Simpson Dominance Index	Margalef Richness Index
JY	2.32 ± 0.64 b	0.49 ± 0.2 b	3.34 ± 0.64 b
YS	2.53 ± 0.05 a	0.51 ± 0.02 ab	3.89 ± 0.09 ab
ZZ	2.59 ± 0.01 a	0.52 ± 0.02 a	4.15 ± 0.13 a

Note: Different lowercase letters indicate significant differences among plots (*p* < 0.05).

**Table 2:** Principal component eigenvalues and variance contribution rates of the indicators in relation to different vegetation types.

Principal Component	Eigenvalue	Variance Contribution Rate/%	Cumulative Variance Contribution Rate/%
PC1	4.99	45.39	45.39
PC2	3.13	28.48	73.87
PC3	1.20	10.92	84.78

**Table 3:** Factor loadings of the indicators on the principal components in relation to different vegetation types.

Indicator	PC1	PC2	PC3
Bulk density	−0.76	−0.36	0.14
Total soil porosity	0.74	0.05	0.54
Capillary porosity	0.79	0.38	−0.36
Non-capillary porosity	−0.60	−0.38	0.63
Maximum water-holding capacity	0.86	0.33	0.00
Capillary water capacity	0.89	0.35	0.20
Field water capacity	0.69	−0.05	0.47
Water content	0.63	−0.59	−0.02
Shannon-Wiener diversity index	−0.36	0.89	0.20
Simpson dominance index	−0.51	0.80	−0.06
Margalef richness index	−0.34	0.85	0.25

**Table 4:** Comprehensive evaluation of eco-hydrological synergistic capacity in relation to different vegetation types.

Plot		Factor Scores of the Principal Components			Membership Function Value			D	Ranking
		FAC1	FAC2	FAC3	PC1	PC2	PC3		
JY	replicate 1	−0.17	0.29	−0.24	0.67	0.72	0.56	0.48	3
JY	replicate 2	−2.28	0.23	−0.56	0.00	0.70	0.47		
JY	replicate 3	−0.61	−1.32	1.23	0.53	0.19	1.00		
YS	replicate 1	0.27	0.69	−0.33	0.81	0.85	0.54	0.82	1
YS	replicate 2	−0.17	0.21	0.28	0.67	0.70	0.72		
YS	replicate 3	0.86	1.13	0.99	1.00	1.00	0.93		
*ZZ*	replicate 1	0.48	0.84	0.68	0.88	0.91	0.84	0.74	2
*ZZ*	replicate 2	0.85	−1.89	0.07	1.00	0.00	0.65		
*ZZ*	replicate 3	0.78	−0.20	−2.12	0.97	0.56	0.00		

**Table 5:** Basic conditions of the sample plots in the study area.

Sample Plot	Tree Height (m)	Diameter at Breast Height (cm)	Planting Spacing (m)	Crown Width (m)	Dead Branch Rate (%)	Stand Density (Stems/ha)	Grazing Status
JY	5.86 ± 1.45	11.25 ± 3.19	/	5.03 ± 1.88	10.66 ± 1.15	60	Severe disturbance
YS	7.43 ± 1.15	17.51 ± 1.69	2 × 5	4.03 ± 1.88	0 ± 0	1000	Light disturbance
ZZ	4.36 ± 0.93	12.42 ± 3.07	5 × 5	3.68 ± 1	1 ± 10.38	400	Light disturbance

## Data Availability

All data in this study can be found in the manuscript.
